# The influence of stigma and disability acceptance on psychosocial adaptation in patients with stoma: A multicenter cross-sectional study

**DOI:** 10.3389/fpsyg.2022.937374

**Published:** 2022-12-08

**Authors:** Zhang Xi, Chen M. Rong, Lin J. Ling, Zeng P. Hua, Gao Rui, Huang G. Fang, Wang Long, Zhuo H. Zhen, Li Hong

**Affiliations:** ^1^Department of Gastroenterology, Fujian Provincial Hospital, Sheng li Clinical Medical College of Fujian Medical University, Fuzhou, China; ^2^Sheng li Clinical Medical College of Fujian Medical University, The School of Nursing, Fujian Medical University, Department of Nursing, Fujian Provincial Hospital, Fuzhou, China; ^3^Graduate School, Fujian Medical University, Nursing School of Fujian Medical University, Fuzhou, China; ^4^Department of Plastic and burn, Fujian Provincial Hospital, Sheng li Clinical Medical College of Fujian Medical University, Fuzhou, China; ^5^Department of Pathology, Fujian Provincial Hospital, Sheng li Clinical Medical College of Fujian Medical University, Fuzhou, China

**Keywords:** stoma, stigma, disability acceptance, psychosocial adaptation, structural equation model

## Abstract

**Background:**

The stoma can cause serious physical and psychological distress to the patient, leading to an inability to live a normal life; although it effectively improves the 5-year survival rate of patients.

**Objective:**

The purpose of this study is to explore the status of stigma and disability acceptance of patients with stoma and their influences on psychosocial adaptation.

**Design:**

A multicenter cross-sectional study.

**Methods:**

A total of 259 patients with stoma in 6 hospitals from southeast China were enrolled. And this research adhered to the STROBE guideline and approved by the Ethics Committee of Fu Jian Provincial Hospital. The ostomy adjustment inventory-20、acceptance of disability scale and social impact scale were used to collect data. The hypothetical path model was tested using the SPSS version 22.0 software and AMOS version 26.0 software.

**Results:**

Stigma, disability acceptance and psychosocial adaptation was associated. The sense of stigma was severe (72.76 ± 12.73), the acceptance of disability was medium (179.24 ± 32.29) and the psychosocial adaptation was poor (38.06 ± 8.76). Also, the hypothesis model of this study fitted the data well (AGFI = 0.967>0.08; *χ*^2^/df = 1.723, *p* = 0.08 > 0.05), and the results showed that disability acceptance positively affected psychosocial adaptation; while stigma negatively affected psychosocial adaptation, and disability acceptance mediated between stigma and psychosocial adaptation (*p* < 0.01).

**Conclusion:**

The stigma and disability acceptance of patients with stoma are serious problems that are closely related to their psychosocial adaptation. Medical staff should take some interventions based on different paths to reduce stoma patients’ stigma and guide them to improve disability acceptance, thus to improve the level of psychosocial adaptation of patients with stoma.

## Introduction

The incidence of colorectal cancer is the third highest worldwide with the second highest mortality, with 1.5 million new cases of colorectal cancer every year and 525 thousand deaths (Siegel et al., 2022). China has a high incidence of colorectal cancer, and the incidence rate and mortality rate of colorectal cancer have increased in recent years (Jie et al., 2019). Surgery is the main treatment for colorectal cancer, and enterostomy is an operation that connects the intestinal lumen with the abdominal wall, which can reduce the threat of tumor to life (Melotti et al., 2013; Yang et al., 2020).

Enterostomy is mainly divided into temporary stoma and permanent stoma according to whether the anus is preserved or not, and the majority of patients in China undergo temporary enterostomy (Ye, 2019). Temporary enterostomy involves lifting the intestinal tube to the abdominal wall as a temporary outlet for human excreta, and it is the first choice for the treatment of rectal cancer and reducing anastomotic leakage (Du, 2020). Patients with temporary stoma undergo enterostomy closure after operations for 3 months or more. However, due to the patient’s own disease progression and willingness, some temporary stoma will evolve into permanent stoma (Keane et al., 2019). Currently, there are nearly 1 million permanent stoma patients in China, and approximately 100,000 new stoma patients are added every year, which is still increasing (Geng et al., 2017). Although enterostomy has prolonged the lives of patients, and the 5-year survival rate has increased to more than 60%, changes in body image, defecation style and lifestyle have caused serious adverse effects on the physical and mental health and quality of life of patients, especially for patients with temporary stoma converted into permanent stoma (Gesaro, 2016; Herrle et al., 2016).

Studies have shown that, in addition to the fear of cancer, patients with stoma cannot tolerate involuntary defecation, intestinal mucosal exposure, stool leakage and the bad smell (Ayaz-Alkaya, 2019). However, to continue life, patients with stoma are forced to accept stomas; after surgery, they usually must face various physiological, psychological and social pressures directly (Chung et al., 2015; Ayaz-Alkaya, 2019). Most patients with stomas have a sense of shame (Phelan et al., 2013; Yuan et al., 2018). In severe cases, they can have social psychological and behavioral problems, such as self-acceptance disorder and social interaction disorder, and cannot return to daily life smoothly (Gesaro, 2016; de Lima et al., 2018; Ayaz-Alkaya, 2019). The original meaning of stigma is brand, which refers to an individual being different due to his or her own disease and being widely rejected and not accepted by the people around him or her; the psychological and social aspects of patients are unhealthy, forming a vicious circle (Fangfang, 2016). Yuan Jing min and other surveys found that 44.3% of patients with permanent colostomies had severe stigma, which led to poor stoma adaptation and decreased quality of life (Yuan et al., 2018; Qin et al., 2020).

Many a studies have shown that the reason for stigma in patients with stoma is the internalization of negative emotions and experience integration (Pasmatzi et al., 2016; Xian et al., 2018). In addition, stomas for patients are not a disease but a disability; it is the destruction of the integrity of the body and psychological trauma (Lack and Notter, 2021). After surgery, patients not only face the lack of physiological function but also bear the impact of body shape changes on their social interaction and family function. Studies have shown that it is difficult for patients with stomas to fully accept a series of changes, such as body changes, and the disability acceptance of patients with stoma is generally low (Mou et al., 2018). The acceptance of disability is the degree of individual acceptance of one’s own disability status, and it is also the process of individual adaptation to one’s own values and social life^.^ Good acceptance by the disabled can change the individual’s actual perception, and the loss of personal value and social value caused by the disability will not have a negative impact on the existing normal ability and the meaning of individual existence (Knowles et al., 2017). Studies have shown that the stigma and disability acceptance of patients with stoma is closely related to their psychosocial adaptation, People with higher disability acceptance have greater quality of life and stronger psychosocial adaptation, which are conducive to the treatment and rehabilitation of patients with stoma (Ran et al., 2016; Zhang et al., 2019). How to reduce the negative emotion of patients with stoma, rationally accept the physical disability, enhance the ability of psychosocial adaptation and improve the quality of life is the direction of medical staff in recent years. However, based on the literature, the current study mainly focused on quality of life and its influencing factors in patients with stoma (Yilmaz et al., 2017; Heins et al., 2020). In addition, studies have shown that social psychological adaptation directly affects the treatment, prognosis and quality of life of patients (Knowles et al., 2013; Wu et al., 2020).

Nevertheless, there have been few studies on the stigma and disability acceptance of patients with stoma especially whether the stigma and disability acceptance of patients with stoma is related to their psychosocial adaptability and whether it affects their daily life. To our best knowledge, the mechanism between psychosocial adaptation and stigma and disability acceptance of stoma patients’ needs to be further discussed. At present, there is no structural equation model study on psychosocial adaptation of stoma patients. Therefore, the medical staff must pay attention to the psychological state of patients with stoma, and take effective measures to improve their quality of life and help them return to society smoothly.

## Objective

**Figure 1:**
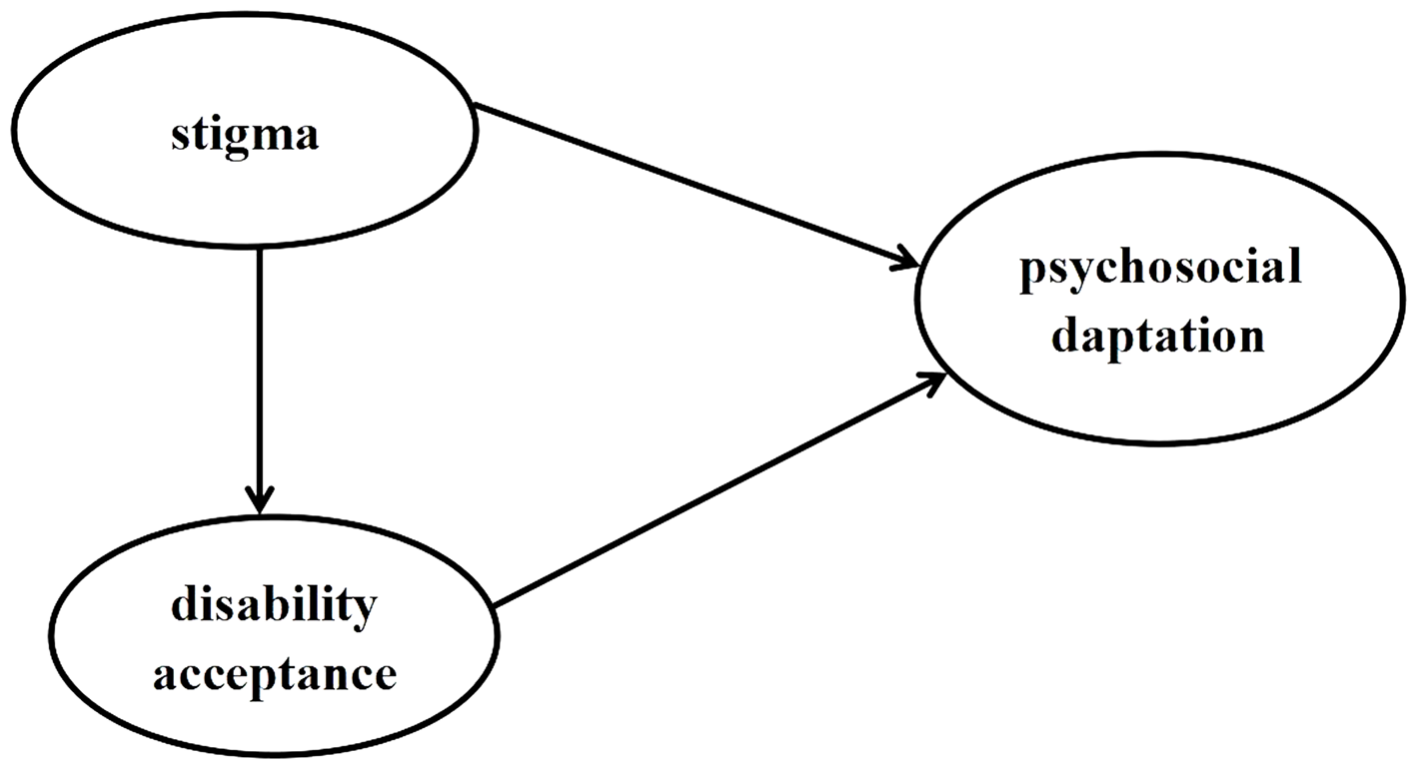
Theoretical model and hypotheses.

The aim of this study is to examine the status of stigma and disability acceptance of patients with stoma and test a hypothetical model that estimates the influence of stigma and disability acceptance on psychosocial adaptation. The following three hypotheses were proposed in this study (Figure 1). The hypothesis are as follows:

1. Stigma has a significant direct effect on psychosocial adaptation.
2. Stigma has a significant direct effect on disability acceptance; disability acceptance has a significant direct effect on psychosocial adaptation.
3. Stigma has a significant indirect effect on psychosocial adaptation *via* disability acceptance.

## Materials and methods

### Design and ethical approval

This study was designed as a cross-sectional descriptive survey and also adhered to the Strengthening the Reporting of Observational Studies in Epidemiology (STROBE) guideline. Besides, the study was conducted in agreement with the Helsinki Declaration and approved by the Ethics Committee of Fu Jian Provincial Hospital (K2019-03-022). Written informed consent to participate in this study was provided.

### Participants

A total of 281 patients with stoma were selected through convenience and snowball sampling. The inclusion criteria were as follows: (1) pathologically diagnosed with colorectal cancer (ICD-10 code C18), and with enterostomy for no more than 6 months; (2) ≥ 18 years old; (3) primary school education or above, with reading and communication skills; (4) stable condition, without serious life-threatening diseases; and (5) voluntary participation in this study. The exclusion criteria were as follows: (1) Cognitive impairment, mental illness history or AIDS history; (2) patients with other types of tumors, tumor recurrence or metastasis; (3) people with disabilities in other parts of the body; (4) those who have participated in similar studies with disabilities in other parts of the body; and (5) those who have participated in similar studies.

### Measurements

#### General demographic data questionnaire

The questionnaire was designed by the research group, which including age, gender, marital status, education level, living area, average income, and the related information of stoma including the types of stoma，duration of stoma (month), the frequency of stool omission and stool leakage and the degree of spouse accepting stoma etc.

#### Social impact scale

This scale was compiled by Fife et al. in 2000 and translated into Chinese by Boyd et al. (2014) and Pan et al. (2007). The SIS includes 4 dimensions and 24 items, namely social exclusion (9 items), economic insecurity (3 items), intrinsic shame (5 items) and social isolation (7 items). Four-point Likert scores were used for the scale. A score of 1–4 points was used to represent extremely agree, agree, disagree and extremely disagree. All of the items were scored in reverse, for a total score of 24–96: 24–47 was a mild level, 48–71 was a moderate level, and 72–96 was a severe level. The higher the score was, the more severe the stigma was. The Cronbach’s α coefficient of the scale was 0.85–0.90, and the correlation coefficient of each dimension was 0.28–0.66. Studies have shown that the SIS can be used to measure the stigma level of patients with enterostomies.

#### Acceptance of disability scale

AODS was developed by Linkowski in 1971 to describe an individual’s attitude toward disability (Linkowski, 1971). In 2010, the Taiwanese scholars Chao et al. translated it into Chinese (Chao et al., 2010). There are 4 dimensions, including the expanding dimension, obedience dimension, control dimension and transformation dimension. Each item adopts a 6-level scoring method, and 15 items are positively scored, i.e., “strongly disagree” to “strongly agree” are scored 1–6, respectively; the remaining 35 items are scored in the opposite fashion. The total score of the AODS was 50–300; 50–133 indicated low acceptance, 134–217 indicated medium acceptance, and 218–300 indicated high acceptance. The Cronbach’s α coefficient of the AODS was 0.910, and the content validity was 0.81.

#### Ostomy adjustment inventory-20

Simmons et al. developed the OAI-20 in 2009 and translated it into a Chinese version in 2011 to evaluate the psychological adaptation levels of patients in the process of returning to society (Simmons et al., 2009; Jun et al., 2011). The scale includes three dimensions and 20 items, namely positive emotion (6 items), negative emotion (5 items) and social life adaptation (9 items). The 5-grade Likert scoring method was used. Positive items were scored positively, and negative items were scored in the opposite manner: total agreement = 4 points, agreement = 3 points, uncertainty = 2 points, disagreement = 1 point, total disagreement = 0 points. The total score of the scale is 0–80 points; ≤ 40 points indicates a low degree of adaptation, 41–59 points indicates moderate adaptation, and ≥ 60 points indicates high adaptation; the higher the score is, the higher the level of social psychological adaptation is. Cronbach’s coefficient for the OAI scale was 0.87, and Cronbach’s coefficient of each dimension was 0.73–0.78.

### Data collection

A unified questionnaire was administered in face-to-face interviews by well-trained interviewers. The survey completed in a quiet environment and the subjects completed the questionnaire in person according to their actual situation. As for those who had difficulty completing it, the investigators assisted them according to the instructions for the research subjects. After the questionnaire was completed, its integrity was checked in a timely manner to ensure the validity and authenticity of the information. If two or more answers were selected for the same question in the questionnaire, the number of unanswered questions for the whole questionnaire was more than two, or the answers for the whole questionnaire were identical, the questionnaire was regarded as invalid. All questionnaires were anonymous. Before the formal investigation, 30 subjects were investigated to understand and be familiar with the whole research process, and problems in the pre-experiment were improved. The research took place in six hospitals during hospitalization and after discharge from July 2019 to November 2020 in southeast China (Table 1). Finally, 12 refused to participate midway, 4 provided the wrong demographic information, and 6 completed the questionnaire incorrectly. two-hundered fifty-nine out of 281 questionnaires are valid and the effective rate was 92.17%.

**Table 1:** The number and percentage of patients with stoma in different hospital (*N* = 259).

**Hospital**		***N***	**%**
**Type of hospital**			
	General hospital	221	85.33
	Specialized hospital	60	23.12
**Hospital level**			
	Level 3-Class A	167	64.48
	Level 3-Class B	79	30.5
	Level 2-Class A	35	13.51

### Statistical methods

SPSS software, version 22.0 (IBM, Armonk, NY, United States) and AMOS version 26.0 software were used for the statistical analysis. The count data was described by frequency and constituent ratio, and the measurement data were described by mean ± standard deviation. The normal distribution samples were tested by using the t-test, and the non-normal distribution was tested by using nonparametric test. Spearman correlation analysis was used to analyze the scores of SIS, AODS and OAI-20. The significance level was set at 0.05. To evaluate the fitness of the hypothetical model, the chi-square/degrees of freedom ratio (*x*^2^/df ), goodness-of-fit index (GFI), root mean square error of approximation (RMSEA), adjusted goodness-of-it index (AGFI), the incremental fit index (IFI), normed fit index (NFI), comparative fit index (CFI) and Tucke-Lewis index (TLI) were used. The following threshold values were recommended as criteria for an adequate model: *x*^2^/df < 3.00, GFI > 0.80, RMSEA <0.08, AGFI >0.80, IFI > 0.90, NFI > 0.90, CFI > 0.90 and TLI > 0.90 (Wu, 2013).

## Results

### Sample characteristics

This study collected 259 patients with stoma. There were more men than women (52.51% vs. 47.49%), most of them were older than 60 years old, and the majority had high school qualifications (34.36%). Most of the patients lived in rural areas, and their family income was 5,000–10,000 yuan per month. The number of temporary stoma was greater than that of permanent stoma (60.62% vs. 39.38%), ileostomy was the most common, and the stoma time was 3–6 months; and 79.53% of the patients with stoma had stool leakage and stool smell leakage. The general demographic data of more patients with stoma are shown in Table 2.

**Table 2:** Sociodemographic and clinical characteristic of the sample (*N* = 259).

**Variables**	***N***	**%**
**Age**		
<45	26	10.04
45–59	101	38.4
≥60	132	50.97
**Gender**		
Male	123	47.49
Female	136	52.51
**Marital status**		
Married	246	94.98
Unmarried	2	0.77
Others^1^	11	4.25
**Education level**		
Primary	63	24.32
Secondary	97	37.45
Tertiary	99	38.22
**Living area**		
Rural area	111	42.86
Urban area	148	57.14
**Average income(RMB/Month)**		
<5,000	76	29.34
5,000–1,000	129	49.81
≥10,000	54	20.85
**Medical insurance type**		
SMI	89	34.36
URMI	132	50.97
NRCMI	38	14.67
**Types of stoma**		
Temporary	157	60.62
Permanence	102	39.38
**Stool omission**		
Never	49	18.92
Sometimes	84	32.43
Always	126	48.65
**Stool leakage**		
Never	53	20.46
Sometimes	81	31.27
Always	125	48.26
**Spouse receiving stoma**		
Yes	33	12.74
No	226	87.26
**Stoma time (Months)**		
≤3	68	26.25
3–6	132	50.97
≥6	59	22.78
**Stoma location**		
Ileostomy	109	42.08
Transverse colostomy	52	20.08
Sigmoid colostomy	98	37.84

^1^Including unmarried, divorced, widows and widowers.

SMI, Staff Medical Insurance; URMI, Urban Residents Medical Insurance; NRCMI, New Rural Cooperative Medical Insurance.

### The comparison of SIS, AODS, and OAI-20 score

In this study, 259 patients with stoma were investigated. The stigma scores ranged from 29 to 87, with a total score of (72.76 ± 12.73). The scores for stigma was severe. The disability acceptance scores of stoma patients ranged from 131 to 273, with a total score of (179.24 ± 32.29), showing a moderate level of disability acceptance. The psychosocial adaptation scores of patients with stoma ranged from 31 to 69, with a total score of (38.06 ± 8.76), showing a low psychosocial adaptation ability. All results are shown in Table 3.

**Table 3:** The comparison of SIS, AODS and OAI-20 score (*N* = 259).

**Variables**	**Total score**	**Range**	**Mean(SD)**
**SIS**	96	24–96	72.76 ± 12.73
Social exclusion	36	18–34	27.63 ± 3.95
Economic insecurity	12	4–10	7.81 ± 2.52
Intrinsic shame	20	12–19	15.87 ± 3.06
Social isolation	28	13–26	21.45 ± 2.89
**AODS**	300	50–300	179.24 ± 32.29
Expanding dimension	84	14–84	58.79 ± 10.86
Obedience dimension	30	5–30	12.65 ± 4.59
Control dimension	96	16–96	56.28 ± 9.63
Transformation dimension	90	15–90	51.52 ± 8.77
**OAI-20**	80	0–80	38.06 ± 8.76
positive emotion	24	0–24	12.51 ± 2.43
negative emotion	20	0–20	11.62 ± 1.98
social life adaptation	36	0–36	13.93 ± 4.05

(1) SD, Standard Deviation. (2) SIS, Social Impact Scale; (3) AODS, Acceptance of disability Scale; (4) OAI-20, Ostomy Adjustment Inventory-20.

### Correlation analysis of SIS, AODS, and OAI-20 score

Pearson’s correlation analysis showed that the score for SIS was negatively correlated with the score for OAI-20 and its factors (*p* < 0.05) and the score for AODS and its factors (p < 0.05). While the score for AODS was positively correlated with the score for OAI-20 and its factors (p < 0.05), as shown in Table 4.

**Table 4:** Spearman correlation coefficients of OAI-20, SIS and AODS Score (*r*, *N* = 259).

**Variables**	**OAI-20 (Total score)**	**OAI-20-1**	**OAI-20-2**	**OAI-20-3**	**SIS1**	**SIS2**	**SIS3**	**SIS4**	**AODS1**	**AODS2**	**AODS3**	**AODS4**
**OAI-20** **(Total score)**	1.00	–	–	–	–	–	–	–	–	–	–	–
OAI-20-1	0.794*	1.00	–	–	–	–	–	–	–	–	–	–
OAI-20-2	0.862*	0.871*	1.00	–	–	–	–	–	–	–	–	–
OAI-20-3	0.831*	0.831*	0.693*	1.00	–	–	–	–	–	–	–	–
**SIS**												
SIS1	−0.541*	−0.673	−0.762*	−0.469*	1.00	–	–	–	–	–	–	–
SIS2	−0.621*	−0.845*	−0.472*	−0.539*	0.243*	1.00	–	–	–	–	–	–
SIS3	−0.477*	−0.504*	−0.696*	−0.628*	0.394*	0.389*	1.00	–	–	–	–	–
SIS4	−0.463*	−0.491*	−0.501*	−0.433*	0.311*	0.458*	0.462*	1.00	–	–	–	–
**AODS**												
AODS1	0.467*	0.585	0.451*	0.687*	−0.382*	−0.629*	−0.471*	−0.737*	1.00	–	–	–
AODS2	0.795*	0.494*	0.697*	0.462*	−0.562*	−0.466*	−0.521*	−0.702*	0.533*	1.00	–	–
AODS3	0.593*	0.462*	0.466*	0.509*	−0.464*	−0.583*	−0.422*	−0.583*	0.462*	0.445	1.00	–
AODS4	0.422*	0.388*	0.502*	0.453*	−0.393*	−0.606*	−0.356*	−0.405*	0.681*	0.704*	0.661	1.00

(1) OAI-20: Ostomy Adjustment Inventory-20; OAI-20-1-3nnnpositive emotion、negative emotion and social life adaptation. (2) SIS: Social Impact Scale; SIS1-4: social exclusion, economic insecurity, intrinsic shame and social isolation. (3) AODS: Acceptance of disability Scale; AODS1-4: expanding dimension, obedience dimension, control dimension and transformation dimension. *p < 0.05.

### Fitness of the hypothetical path model

**Figure 2:**
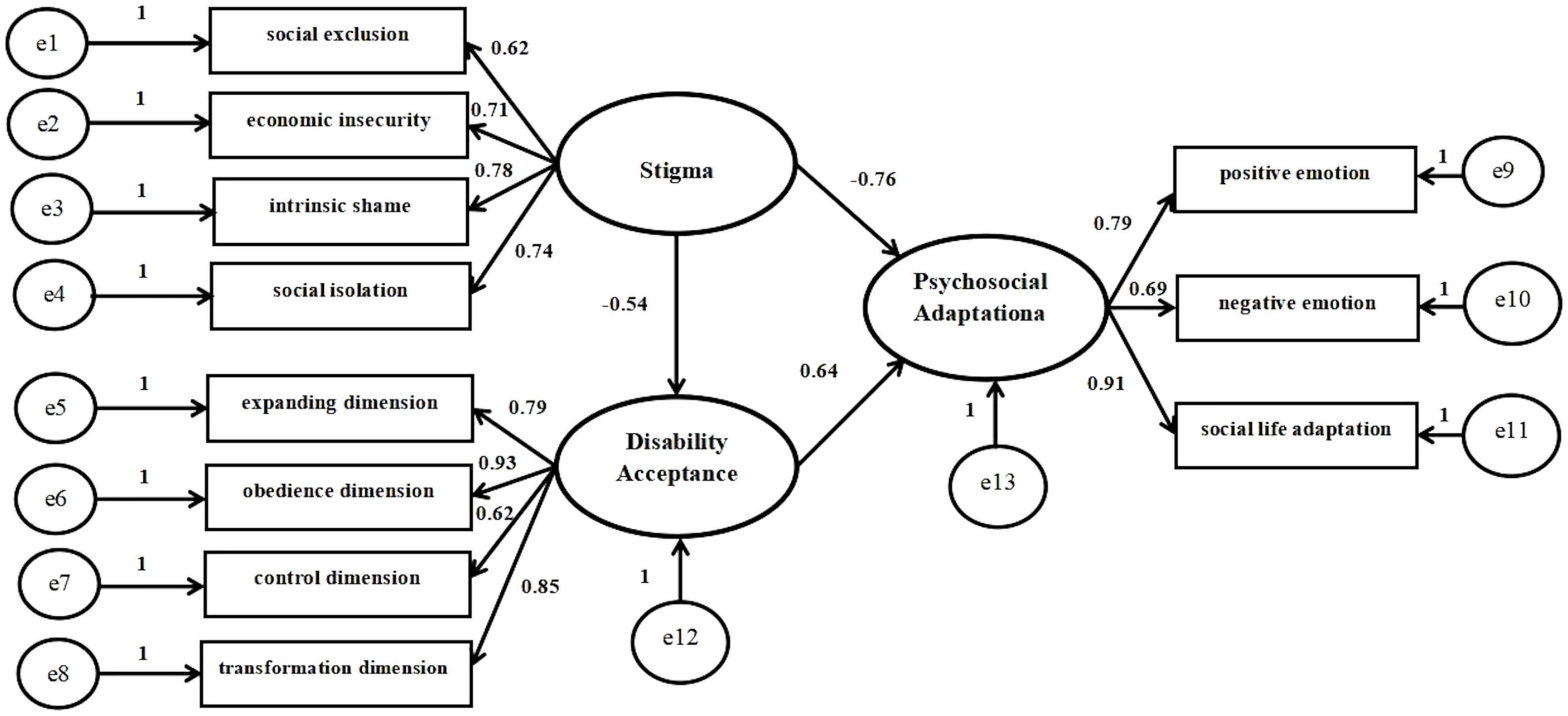
Structural equation model of stigma, disability acceptance and psychosocial adaptation in patients with stoma.

Based on correlation analysis, we used structural equation model to further analyze the data, which is shown in Figure 2. The model parameters are estimated by maximum likelihood method, and the modified structural model *χ*^2^/df = 1.723, *p* = 0.08 > 0.05,within the range of adaptation standard 1 ~ 3. The GFI, AGFI, NFI, IFI, TLI, and CFI are above 0.90, which meets the adaptation standard. With RMSEA = 0.071, which meets the adaptation standard less than 0.08, as shown in Table 5. The above indicators are up to standard, which proves that the model is well adapted. At the same time, the three assumptions were supported in the model. It was found that stigma has a significant direct effect on psychosocial adaptation (*β* = 0.542, *p<*0.01, 95% confidence interval (95%CI) [−0.615, −0.273]. Stigma has a significant direct effect on disability acceptance; disability acceptance has a significant direct effect on psychosocial adaptation (*β* = −0.342, *p<*0.01, 95% confidence interval (95%CI) [−0.575, −0.181]; *β* = 0.465, *p<*0.01, 95%CI [0.241, 0.735]). Furthermore, Stigma has a significant indirect effect on psychosocial adaptation *via* disability acceptance (*β* = 0.053, *p<*0.01, 95% confidence interval (95%CI) [0.028, 0.125]. Table 6 represents the specific effect values of each path in this model. Therefore, the hypothesis model of this study fitted the data well, and the results showed that disability acceptance positively affected psychosocial adaptation, while stigma negatively affected psychosocial adaptation. It was also found that disability acceptance mediated between stigma and psychosocial adaptation.

**Table 5:** Fitting index of the relationship model between SIS, AODS. and OAI-20.

Estimate	Model fitting value	Standard
χ^2^/df	1.723	< 3.00
GFI	0.936	>0.80
AGFI	0.967	>0.80
TLI	0.953	>0.90
CFI	0.972	>0.90
IFI	0.925	>0.90
NFI	0.934	>0.90
RMSEA	0.071	<0.08

GFI-goodness-of-fit index; AGFI -adjusted goodness-of-it index; TLI-Tucke-Lewis index; CFI-comparative fit index; IFI-incremental fit index; NFI-normed fit index; RMSEA-root mean square error of approximation.

**Table 6:** The direct effects, indirect effects, and total effects of each path in the model.

**Estimate**	***β***	**BC 95%-C I**^**a**^		***p***	**Lower**	**Upper**
**Direct effects**				
SIS→OAI-20	−0.542	−0.615	−0.273	<0.01
AODS→OAI-20	0.465	0.241	0.735	<0.01
SIS→AODS	−0.342	−0.575	−0.181	<0.01
**Indirect effects**				
SIS→AODS→OAI-20	0.053	0.028	0.125	<0.01
**Total effects**				
AODS→OAI-20	−0.431	0.295	0.736	<0.01
SIS→AODS	−0.319	−0.461	0.070	<0.01

^a^ Means that 95% bias-corrected bootstrap confidence interval.

## Discussion

Stigma can directly predict the psychosocial adaptation of patients with stoma.

Our study showed that the stigma of patients with stomas was at a severe level, similar to the results of Xian et al. (2018), Yuan et al. (2018), and Man-yun et al. (2020). And the study also found the total score and dimensions of stigma of patients with stoma are negatively correlated with their psychosocial adaptation, that is, the higher the stigma, the worse the psychosocial adaptation of patients with stoma. Enterostomy results in body image disorders, possible exhaust noises, and odor and fecal leakage, which cause patients to feel ashamed. And stoma is too obvious to conceal and destroys patients’ fragile self-esteem. After surgery, patients with stoma not only have to face the challenge of cancer, but also need to learn the nursing skills of stoma and they are also under the pressure of returning to social life, which makes the psychological burden of patients with stoma heavy (Gesaro, 2016), Also the inflammatory reaction after surgery further aggravates the psychological pressure (Busetto et al., 2014). In addition, loneliness renders the negative emotions of patients increasingly serious. The scores of internal shame and social isolation in the dimensions of shame were higher in this study, similar to the results of related study (Ernst et al., 2017; Yuan et al., 2018). Internal shame is an emotion that causes individuals to hide their own diseases and not tell others after they have experienced exclusion and economic insecurity (Ayaz-Alkaya, 2019). The patients’ internal shame is further aggravated by the limited life and work caused by diseases and enterostomies. The stigma experienced by stoma patients may be explained not only by the stoma itself but also by the unique cultural characteristics of China. Patients have difficulty accepting the reality of having a stoma in China, and a similar situation occurred in the community surrounding them. Patients with stoma might be misunderstood and unaccepted by colleagues, friends and relatives, which further aggravates the decrease of psychosocial adaptability of patients with stoma (Lim et al., 2015). And the odor and excrement from the stoma greatly affect communication between patients and other people. Decreasing social intercourse renders social isolation more obvious. However, the above risk factors lead to stoma patients feel rejected because of their body image loss, reduced self-esteem, and sense of shame. Thus, patients cannot adapt to the stoma, interfere with their psychosocial adaptation, and cannot smoothly integrate into the new life.

The disability acceptance of patients with stoma can directly predict their psychosocial adaptation. Disability acceptance is the primary factor of psychological adjustment of disabled patients, which is closely related to patients’ healthy psychology and quality of life. Our study found that the disability acceptance of patients with stoma was medium, consistent with the results of Zhao et al. (2013), and Liu et al. (2021). Stoma changes the original appearance and excretion of patients. Some patients believe that they are dirty and unattractively disabled. Stoma for patients is not a disease but a disability. Stoma is damage to the integrity of the body and psychological trauma, and postoperative patients not only face the lack of physiological function, but they also bear the impact of body image changes on family function and social interaction (Ayik et al., 2019). What is more, in the four dimensions of the disability acceptance scale, the score of the obedience dimension was the lowest, especially for individuals who do not care about the physical function and appearance problems caused by disability. The reason might be that the body image is an individual’s perception of his or her own body function and appearance. Stoma destroys the integrity of the patient’s body and causes the patient to feel incomplete, making it difficult for the patient to accept physiological changes in a short time. Because the Stoma changes the original appearance and excretion mode of patients, some patients with stoma regard themselves as unattractive and dirty disabled, especially young and middle-aged patients (Black et al., 2021). In the process of postoperative psychological adjustment, how to accept the change of body image is the biggest challenge for patients with stoma; and then it may trigger a series of negative effects. Research by Crocker and Salomé GM showed that patients who do not accept disability often lead to ineffective psychological regulation and affect their physical and mental health; at the same time, it affects the return to social life (Crocker, 1999; Salomé et al., 2015).

Enterostomy is a common operation for the treatment of colorectal cancer. Although enterostomy is of great significance for the treatment of the disease, it has a serious impact on the physical, psychological and social aspects of patients, especially the patients with permanent stoma (Occhipinti et al., 2015; Mou et al., 2018). Social psychological adaptation involves individuals adjusting their emotions to suit their own situations and to maintain the best psychological state and a smooth return to social life. Studies have shown that, after enterostomy, patients have negative emotions, such as nonacceptance, inferiority complex, anxiety, stigma and even think they are disabled, which affects their social lives. They must be adjusted to achieve social psychological adaptation (Gautam and Poudel, 2016; Song et al., 2020). Our study further confirmed the relationship between stigma, disability acceptance and psychosocial adaptation in patients with stoma by structural equation model (*χ*^2^/d *f* = 1.723; RMSEA = 0.071 < 0.08). In addition to finding that stigma has a significant direct effect on psychosocial adaptation and disability acceptance, disability acceptance has a significant direct effect on psychosocial adaptation in the study. We also found that disability acceptance mediated between stigma and psychosocial adaptation. The negative emotions of patients with stoma, such as shame and low-level disability acceptance, are great challenges for medical staff to help patients with stoma to establish good psychosocial adaptability. As patients with stoma, they have to face not only the blow of cancer, but also the change of body image and defecation pathway; the physical and psychological burden is heavy. While, as medical staff, the ultimate purpose of any treatment measures that we take is to help patients overcome the disease, improve the quality of life, rebuild life hope and smoothly return to social life. This suggests that in the clinical practice, medical staff must pay attention to the psychological state of patients with stoma, encourage them to communicate more with their family and friends, strengthen health education, help them and their family members establish a correct understanding of stoma, reduce their sense of shame, and enable them to accept stoma, adapt to stoma and accept disability rationally. Moreover, according to the patients’ own characteristics and existing problems, targeted intervention measures are taken to help patients with stoma establish good psychosocial adaptation as soon as possible, improve the quality of life and return to society smoothly.

### Study limitations and advantages

The limitations of this study are time and manpower constraints; the sample size and survey area are limited. Nevertheless, our study still has the following advantages: (1) a population-based, multicenter case study was conducted through convenience and snowball sampling, the sample was representative; (2) reinvestigation before the study and strict quality control during the study can reduce the bias of the research results; (3) this study was the first to explore the status and correlation of the stigma, disability acceptance and psychosocial adaptation of patients with stoma by the structural equation model, providing a reference for future clinical work to a certain degree.

## Conclusion

In summary, this study found that the stigma and disability acceptance of patients with stoma are serious problems that are closely related to their psychosocial adaptation. Medical staff should take some interventions based on different paths to reduce stoma patients’ stigma and guide them to improve disability acceptance, thus to improve the level of psychosocial adaptation of stoma patients. Since the manpower and time constraints, we should expand the sample size and area in the future to further confirm the research results.

### Clinical implications

Stoma is of great significance to the treatment of colorectal cancer, but it has a serious impact on the physiological, psychological and social aspects of patients. Unlike other cancer patients, patients with stoma not only face disease threat, but also face the change of body image. Patients have a strong sense of stigma, low disability acceptance and poor psychosocial adaptability. The clinical medical staff should take multi-channel, multi-level and targeted health education measures in and out of hospital to treat the diseases and daily life correctly and reduce the stigma, which gradually help them accept the stoma, and improve the psychosocial adaptation, especially the young patients with stoma; Thus, they can improve the self-care ability, quality of life and return to the society as soon as possible.

## Data availability statement

The original contributions presented in the study are included in the article/supplementary material, further inquiries can be directed to the corresponding author.

## Ethics statement

The studies involving human participants were reviewed and approved by the Ethics Committee of FuJian Provincial Hospital (K2019-03-022). The patients/participants provided their written informed consent to participate in this study.

## Author contributions

All the authors contributed to the article (such as: design, data collection, and data analysis etc.) and approved the submitted version.

## Funding

This Study was supported by the Fujian Provincial Health Technology Project, National Key Clinical Specialty Discipline Construction Program of China (grant number: [2010]313), Sponsored by Fujian provincial Health Technology Project (grant number: 2018-1-27) and Qi Hang of Fu Jian Medical University (grant number: 2020QH1146).

## Conflict of interest

The authors declare that the research was conducted in the absence of any commercial or financial relationships that could be construed as a potential conflict of interest.

## Publisher’s note

All claims expressed in this article are solely those of the authors and do not necessarily represent those of their affiliated organizations, or those of the publisher, the editors and the reviewers. Any product that may be evaluated in this article, or claim that may be made by its manufacturer, is not guaranteed or endorsed by the publisher.
